# Epitope mapping of recombinant *Leishmania donovani* virulence factor A2 (rec*Ld*VFA2) and canine leishmaniasis diagnosis using a derived synthetic bi-epitope

**DOI:** 10.1371/journal.pntd.0005562

**Published:** 2017-05-30

**Authors:** Thais Melo Mendes, Eric Henrique Roma, Fernanda Costal-Oliveira, Lucas de Carvalho Dhom-Lemos, Cristina Monerat Toledo-Machado, Oscar Bruna-Romero, Daniella Castanheiras Bartholomeu, Ricardo Toshio Fujiwara, Carlos Chávez-Olórtegui

**Affiliations:** 1 Departamento de Bioquímica e Imunologia, Instituto de Ciências Biológicas, UFMG, Belo Horizonte—Minas Gerais, Brazil; 2 Departamento de Parasitologia, Instituto de Ciências Biológicas, UFMG, Belo Horizonte—Minas Gerais, Brazil; 3 Departamento de Microbiologia, Imunologia e Parasitologia, CCB, UFSC, Florianópolis–Santa Catarian, Brazil; Institut Pasteur de Tunis, TUNISIA

## Abstract

**Background:**

Leishmaniasis is one of the most important zoonotic diseases spread in Latin America. Since many species are involved in dog infection with different clinical manifestations, the development of specific diagnostic tests is mandatory for more accurate disease control and vaccine strategies.

**Methodology/Principal findings:**

Seventy-five 15-mer peptides covering the sequence of recombinant *Leishmania donovani* virulence factor A2 (rec*Ld*VFA2) protein were prepared by Spot synthesis. Membrane-bound peptides immunoreactivity with sera from dogs immunized with rec*Ld*VFA2 and with a specific anti-rec*Ld*VFA2 monoclonal antibody allowed mapping of continuous B-cell epitopes. Five epitopes corresponding to the N-terminal region of rec*Ld*VFA2 (MKIRSVRPLVVLLVC, RSVRPLVVLLVCVAA, RPLVVLLVCVAAVLA, VVLLVCVAAVLALSA and LVCVAAVLALSASAE, region 1–28) and one located within the repetitive units (PLSVGPQAVGLSVG, regions 67–81 and 122–135) were identified. A 34-mer rec*Ld*VFA2-derived bi-epitope containing the sequence MKIRSVRPLVVLLVC linked to PLSVGPQAVGLSVG by a Gly-Gly spacer was chemically synthesized in its soluble form. The synthetic bi-epitope was used as antigen to coat ELISA plates and assayed with dog sera for in vitro diagnosis of canine visceral leishmaniasis (CVL). The assay proved to be highly sensitive (98%) and specific (99%).

**Conclusions/Significance:**

Our work suggests that synthetic peptide-based ELISA strategy may be useful for the development of a sensitive and highly specific serodiagnosis for CVL or other parasitic diseases.

## Author summary

Leishmaniasis is a neglected tropical disease being among the six endemic prioritized diseases in the world. Visceral leishmaniasis (VL) is caused by *Leishmania infantum* and represents a serious public health problem in Brazil. Dogs are the main source of infection in the urban area and, in Brazil, the main strategies of the Visceral Leishmaniasis Control Program are directed to control the canine reservoir (serological survey and euthanasia of dogs which present reactive serum). In general, diagnosis of canine visceral leishmaniasis (CVL) has been presented as a problem for Brazilian public health services. The issue should be attributed mainly to the following factors: 1- range of similar clinical signs observed in other infectious diseases that affect dogs; 2- large percentage of asymptomatic or oligosymptomatic dogs; 3- nonspecific histopathological changes; 4- nonexistence of a diagnostic test 100% specific and sensitive. In this work, we developed a synthetic bi-epitope peptide as an antigen for immunodiagnostic ELISA to detect CVL. The biepitope used for ELISA assay accurately distinguish (98% sensitivity and 99% specificity) CVL dogs sera from non-infected dogs sera.

## Introduction

Visceral leishmaniasis (VL) is an infection caused by various species of *Leishmania*, an intracellular protozoan parasite. Currently, VL is among the six endemic prioritized diseases in the world [1]. In humans, infection with *Leishmania* can cause a broad spectrum of symptoms ranging from a clinically silent infection to a fatal visceral disease [2]. In an urban environment, dogs are the main reservoir of the disease, but many stay asymptomatic, showing no clinical signs [3, 4, 5].

A2 is a stress response protein from *L*. *donovani* and it is expressed in amastigote and in promastigote cultures. It corresponds to the specific virulence factor (*Ld*VFA2) and has been shown to be required for *L*. *donovani* amastigote survival in visceral organs of mice [6, 7, 8]. A2 proteins are composed mostly of a variable number of 10-amino-acid repeats and their molecular weight varies from 45 to 100 kDa [9]. *Ld*VFA2 antigens, administered as recombinant protein (rec*Ld*VFA2) or DNA, are protective against *L*. *donovani*, *L*. *amazonensis* and *L*. *chagasi* infections in mice [10, 11, 12], dogs [13] and macaques [14]. Anti- *Ld*VFA2 antibodies have been detected in sera samples from human patients with active visceral leishmaniasis, confirming that *Ld*VFA2 proteins are expressed during infection [10, 15]. These findings suggest that studies of *Ld*VFA2 proteins antigenic properties might have great potential for the development of vaccines, therapeutics and diagnostics for leishmaniasis.

In this work, we report the mapping of B-cell continuous epitopes of rec*Ld*VFA2, production by chemical synthesis of a rec*Ld*VFA2-derived synthetic epitope and its use as antigen for canine visceral leishmaniasis (CVL) diagnosis. Epitope mapping was achieved by peptide-scanning of the rec*Ld*VFA2 sequence using the Spot-synthesis technique [16]. This method is an easy and very flexible technique for simultaneous parallel peptides chemical synthesis on membrane supports. Furthermore, it allows a rapid and low-cost access to a large number of peptides for systematic epitope analysis [17]. Sixty-five overlapping peptides (15-mer frameshifted by 3 residues) covering the complete amino acid sequence of rec*Ld*VFA2 were synthesized on cellulose membranes. Five continuous epitopes corresponding to the non-repetitive N-ter region of rec*Ld*VFA2 (MKIRSVRPLVVLLVC, RSVRPLVVLLVCVAA, RPLVVLLVCVAAVLA, VVLLVCVAAVLALSA and LVCVAAVLALSASAE, region 1–28) were mapped using anti- rec*Ld*VFA2 dog sera and one epitope was located within the repetitive units (PLSVGPQAVGLSVG, region 67–81 and 122–135) using an anti-rec*Ld*VFA2 mAb. An epitope from N-ter (MKIRSVRPLVVLLVC) and another from the C-ter part (PLSVGPQAVGLSVG) were selected and chemically assembled in tandem, to yield a soluble bi-epitope peptide. The bi-epitope used as coating antigen in ELISA accurately distinguish (high sensitivity and specificity) sera of CVL dogs from sera of non-infected dogs.

## Methods

In order to discover a new antigen for use in CVL diagnostics tests, dog’s polyclonal and monoclonal anti-sera *Ld*VFA2 were used to map epitopes. An initial screening was made to select polyclonal antisera with high affinity for *Ld*VFA2 protein and low affinity for the crude extract of *Leishmania infantum*. The epitopes mapped to the selected polyclonal sera and with the specific monoclonal sera were analyzed and two were selected and synthesized as a bi-peptide. This bi-epitope peptide was called rec*Ld*VFA2 derived and it was tested as antigen for CVL diagnosis. The choice of *L*. *donovani* protein for epitope search for use in *L*. *infantum* diagnostic test is due to extensive research on this protein (recVFA2), amino acid sequence availability and biological findings indicating that there is interspecific cross-reactivity.

### Ethics statement

Approval to use the sera samples was obtained from the Committee on Ethics of Animal Experimentation (CETEA, national guidelines Lei 11.794, de 8 de outubro de 2008) from this UFMG (CETEA–protocol 44/2012).

### Production *leishmania* (leishmania*) infantum* antigen *(Li*A*)* and rec*Ld*VFA2

*L*. *infantum* (MHOM/BR/1975/BH46) was grown at 24°C in Schneider´s medium (Sigma, St. Louis, MO, USA) supplemented with 20% heat-inactivated fetal bovine serum (FBS; Sigma), 200 U/mL penicillin and 100 μg/mL streptomycin, at pH 7.2. Total soluble antigens of *L*. *infantum* (*Li*A) was prepared from stationary phase promastigotes, submitted to 7 cycles of freezing (liquid nitrogen) and thawing (42°C), followed by ultrasonication (Ultrasonic processor, GEX600), with cycles of 10 sec for 2 min at 35 MHz. Extracts were then submitted to centrifugation at 8000 × *g* for 20 min at 4°C. The supernatant was collected and stored at −70°C. rec*Ld*VFA2, a recombinant form of A2, was expressed and purified as previously described [15].

### Dog sera and monoclonal antibody against rec*Ld*VFA2

For epitope mapping, sera from seventy-three dogs immunized with rec*Ld*VFA2 were obtained from the laboratory HERTAPE-KALIER Health Animal S.A. For diagnostic test, serum of dogs from a CVL non-endemic area and giving negative results for *L*. *infantum* in immunofluorescence antibody test (IFAT) and confirmed by parasitological test and microscopic analysis of bone marrow aspirates were considered to be non-infected and used as the control group (NI, n = 101). *Leishmania*-infected dog sera (I, n = 101) were obtained from an endemic area for CVL in the Minas Gerais State of Brazil. Infection status was determined by parasitological test, the positivity was confirmed by microscopic analysis of bone marrow aspirates. The positive and negative status was further confirmed by real time PCR. Samples from dogs experimentally infected with *Trypanosoma cruzi* (TC, n = 10), but parasitologically negative for *Leishmania*, were included in this study to evaluate possible cross-reactivity. All sera used for diagnostic test were obtained from sera bank already existing of Laboratory of Immunology and Genomics of Parasites, Federal University of Minas Gerais (UFMG/BR). The anti- *L*. *donovani* virulence factor A2 murine monoclonal antibody (mAb-anti rec*Ld*VFA2) was kindly provided by Dr. Greg Matlashewski, McGill University, Quebec, Canada.

### Identification new antigen for CVL diagnostic

#### Selection of polyclonal sera by ELISA

Falcon flexible microtitration plates (Becton Dickinson France S.A.) were coated overnight at 5^°^C with 100 μl of a 10 μg/ml solution of *Li*A and 2 μg/ml solution of rec*Ld*VFA in 0.02M sodium bicarbonate buffer, pH 9.6. Plates were washed with PBS 1x, 0.1% Tween 20 (v/v) and then blocked with PBS 1x, 0.1% Tween 20 and 2% caseine (w/v) for 1 h at 37°C. Anti-rec*Ld*VFA dog sera (diluted 1:100) were added to respective wells. Plates were incubated for 2 h at 37°C. Binding was detected using an anti-Dog IgG peroxidase conjugated (Sigma) diluted 1:5000 in blocking buffer. After 1 h at 37°C and washing, the peroxidase substrate was added. The reaction was stopped with H_2_SO_4_ 1N after 30 minutes and the resulting colour was measured at 492 nm with an automated microtiter plate reader (Model 450, Bio-Rad). All measurements were made in triplicate.

### B-cell epitope mapping of rec*Ld*VFA

#### Spot peptides synthesis on cellulose membrane

Seventy-five 15-mer overlapping peptide frameshifted by three amino acids derived from the sequence of A2 protein (GenBank accession number: AAB30592.1) were synthesized on a cellulose membrane (Intavis, Koln, Germany) according to [18] and an ASP222 robot (Intavis) was used for the coupling steps. N-terminal acetylation of the peptides was also performed to increase their stability. After peptide sequences had been assembled, the side-chain protecting groups were removed by trifluoracetic acid treatment as described before [19].

#### Immunoassay with cellulose membrane-bound peptides

After an overnight saturation step with blocking buffer (Genosys, France), the set of membrane bound peptides was probed by incubation with serum from four dogs immunized with recA2p (diluted 1:100) for 2 h. Polyclonal antibodies binding was detected by using an alkaline phosphatase-conjugated anti-dog antibody (Sigma) diluted 1:2000 for 1 h, and by the addition of a phosphatase substrate [5-bromo-4-chloro-3-indolyl phosphate (BCIP) and 3-(4,5-dimethylthiazol-2-yl)-2,5-diphenyltetrazolium bromide (MTT) (Sigma), which generates a blue precipitate over spots harboring a peptide recognized by antibodies. A similar procedure was adopted for assays with mAb, used at 2.0 μg/ml. In this case, peptide reactivity was revealed by using an alkaline phosphatase-conjugated anti-mouse antibody (Sigma) diluted 1:2000. To allow its reuse, the membrane was sequentially treated with dimethylformamide, 1% SDS, 0.1% 2-mercaptoethanol in 8 M urea, ethanol/water/acetic acid (50:40:10 vol/vol/vol) and, finally, methanol to remove the precipitated dye and molecules bound to the peptides. The intensity of spots colors were arbitrary quantified using ImageJ software (NIH, Bethesda, MD, USA). The highest reactive spot for each type of epitope mapping (spot 3, for mapping using polyclonal immunized dog sera and spot 48 for mapping using mAb) was considered as presenting 100% of reactivity. The reactivity of all other spots was expressed as percentage of values according to the highest reactive spots.

#### Bi-epitope synthesis of rec*Ld*VFA2 derived

Based on the results of peptide array immunoassay, it was prepared a 34-mer peptide containing two sequences of fifteen amino acids residues in tandem: sequence PLSVGPQAVGLSVG from repetitive part of A2 protein was linked by a Gly-Gly dipeptide to the 15-mer sequence MKIRSVRPLVVLLVC from the non-repetitive part, in order to bring together regions that are apart from each other in the linear sequence of the target peptide. With the purpose of facilitate subsequent affinity based purification of the anti-peptide antibodies, two amino acid residues were added, Lys to the N-ter and Cys to C-ter extremities, respectively. The 34-mer peptide was synthesized manually by F-moc chemistry on a Wang resin (Novabiochem) as described [20] adapted by [21]. After synthesis the protecting groups of side chains were deprotected and the peptide was released from the resin by using TFA in the presence of appropriate scavengers. Then, the peptide was lyophilized and its mass confirmed by mass spectrometry. The synthetic bi-epitope rec*Ld*VFA2 derived (20 mg) was diluted in 1 ml of PBS and used as antigenic preparation to coat ELISA plates. A non-related peptide CRCKPDQGRLRCGYK (nrpep) was also synthesized to be used as negative control.

### Diagnosis of canine leishmaniasis using synthetic bi-epitope

One hundred and one sera from infected dogs were used (I), as well as 101 sera from dogs without a history of *Leishmania* infections (NI) and 10 sera from dogs experimentally infected by *T*. *cruzi* (TC), to verify if the bi-epitope recLdVFA2 derived is a good candidate to be antigen in CVL diagnostic test. Maxisorb flexible microtitration plates were coated overnight at 5°C with 100 μL of synthetic peptide solution (10 μg/mL) in 0.02 M sodium bicarbonate buffer, pH 9.6. Assays were performed as previously described [22]. Sera were diluted 1:100 and absorbance values were determined at 492 nm with a Titertek Multiscan spectrophotometer. All measurements were made in triplicate. Standard EIE-LCV kit for the leishmaniasis diagnosis was used for comparison. This test is the most used in the clinical and serologic testing ELISA for LCV is a good test for use in the field epidemiological serum screening due to its convenience and low cost.

### Statistical analysis

All data comparisons were tested for significance by using unpaired Student’s *t* test or Kruskal–Walls test. Differences were considered statistically significant when *P* values were < 0.05. The lower limit of positivity (cut-off) for bi-epitope and EIE-LCV was established for optimal sensitivity and specificity using the Receiver Operator Curve (ROC curve). The cut-off was chosen based on the point that provides the maximum of the sum of the sensibility and specificity [23]. The performance of each test was evaluated according to the sensitivity (Se), specificity (Sp), positive predictive value (PPV), negative predictive value (NPV), area under the curve (AUC) and accuracy (ACC).

Statistical analyses were performed using GraphPad Prism (version 5.0) and R package for Windows (www.r-project.org) (version 3.1.0).

## Results

### Identification new antigen for CVL diagnostic

#### Selection of dog sera for B-cell epitope mapping

A study with 73 sera from dogs immunized with rec*Ld*VFA2 protein was carried using ELISA to select sera for epitope mapping purposes. Data showed in Table 1 indicate that anti-recVFA2 antibodies were present in all tested sera samples (100%). ELISA values (Abs = 492 nm) varied between 0.195 and 1.4, with an average value of 0.721 ± 0.240. Anti- *Li*A antibodies from dogs immunized with rec*Ld*VFA2 were also measured. Sixty-nine of 73 sera samples, (94.5%) also exhibited cross-reactivity with *Li*A. ELISA values (Abs = 492 nm) varied between 0.055 and 1.10, with an average value of 0.410 ± 0.230. Sera from dogs immunized with rec*Ld*VFA2 which presented very low cross-reaction against *Li*A (Abs values less than 0.10) were used for the epitope mapping of rec*Ld*VFA2. Based in these criteria, sera from 4 dogs with highest ratio between the reactivity for rec*Ld*VFA2 and *Li*A were selected.

**Table 1:** ELISA reactivity of 73 sera of dogs immunized with rec*Ld*VFA2 against rec*Ld*VFA2 and *LiA*.

Dog serum number	Reactivity against		Dog serum number	Reactivity against	
	rec*Ld*VFA2	*Li*A		rec*Ld*VFA2	*Li*A
1	0.771	0.309	38	0.395	0.209
2	0.941	0.382	39	0.661	0.45
3	0.589	0.234	40	0.973	0.365
4	0.832	0.327	41	1.331	0.426
5	0.548	0.294	42	1.077	0.342
6	0.282	0.239	43	0.761	0.336
7	0.939	0.257	44	0.891	0.356
**8**	0.747*	0.069*	**45**	0.868*	0.065*
9	0.644	0.214	46	0.802	0.218
10	0.649	0.255	47	0.949	0.349
11	0.602	0.38	**48**	1.332*	0.095*
12	0.583	0.755	49	0.541	0.711
13	0.678	0.162	50	0.328	0.798
14	0.502	0.573	51	0.794	0.736
15	0.549	0.753	52	0.86	0.341
16	0.715	0.566	53	0.697	0.69
17	0.892	0.956	54	0.554	0.466
18	0.430	0.584	55	0.500	0.25
19	0.339	0.709	**56**	1.228*	0.095*
20	0.804	0.302	57	0.623	0.586
21	0.351	0.346	58	0.729	0.627
22	0.504	0.186	59	0.445	0.446
23	0.932	0.327	60	0.58	0.36
24	1.4	0.631	61	0.571	0.847
25	0.853	0.31	62	0.574	0.646
26	0.748	0.317	63	0.439	0.455
27	0.839	0.92	64	0.594	0.18
28	1.116	1.11	65	0.642	0.288
29	0.712	0.293	66	0.522	0.358
30	0.622	0.846	67	0.195	0.200
31	0.729	0.509	68	0.61	0.449
32	0.858	0.25	**69**	0.767*	0.056*
33	0.785	0.278	70	0.781	0.256
34	0.72	0.385	71	0.996	0.267
35	1.033	0.486	72	0.659	0.055
36	0.733	0.38	73	0.74	0.300
37	0.688	0.369

ELISA plates were coated with of *Li*A and rec*Ld*VFA in 0.02M sodium bicarbonate buffer. Dog sera anti-rec*Ld*VFA were diluted 1:100 were added to respective wells. Peroxidase conjugated anti-IgG dog (Sigma) was 1:5000. The reactivity was measured at 492 nm with a microtiter plate reader (Model 450. Bio-Rad). All measurements were made in triplicate.

*Bold numbers indicate sera that gave a good reactivity response with the rec*Ld*VFA and a negative response with *Li*A.

#### Identification of B-cell epitopes of rec*Ld*VFA2

**Fig 1:**
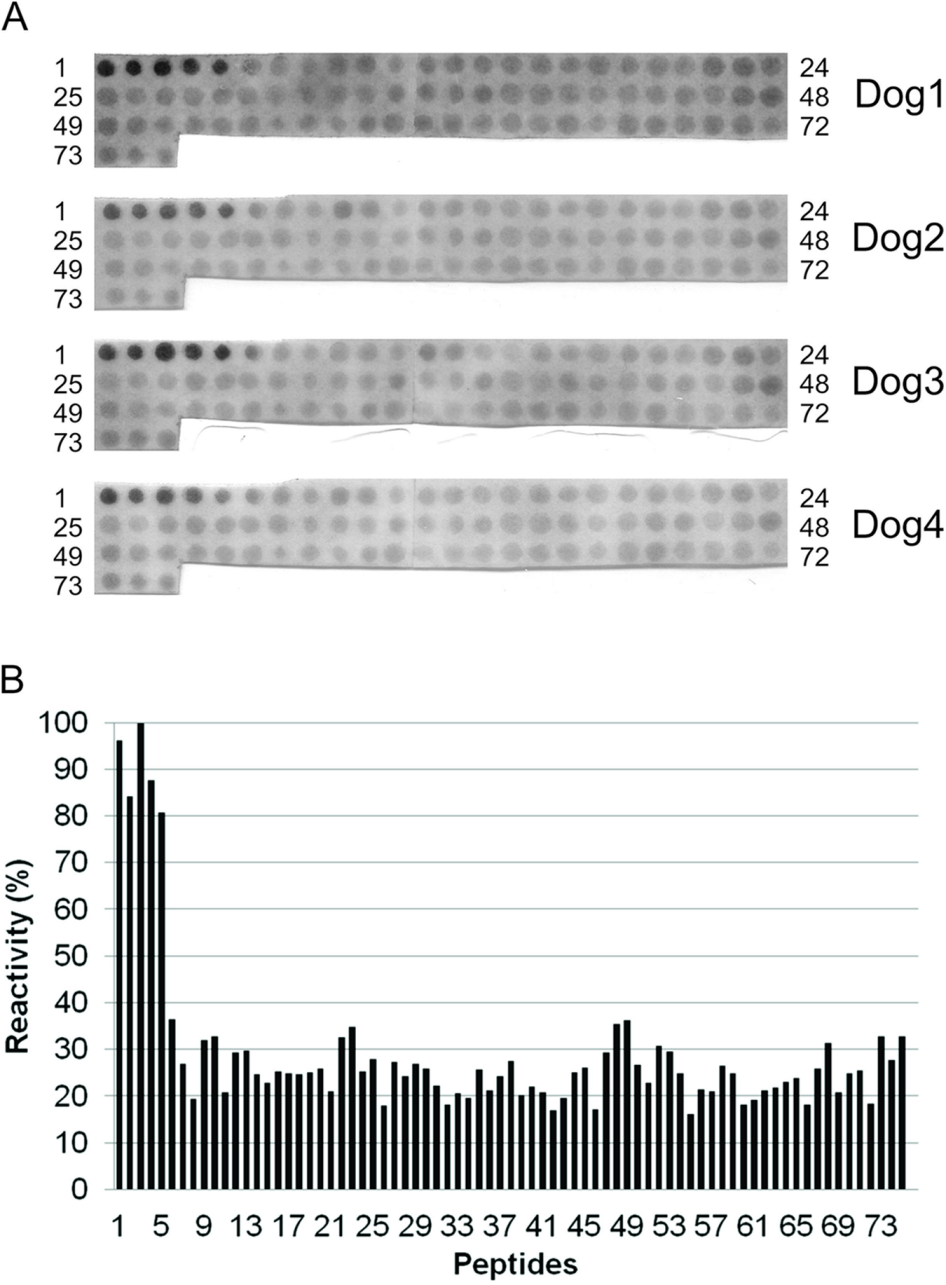
Binding pattern of a dog policlonal antibodies with the overlapping peptides. (A) Reactivity of 15-mer overlapping peptides derived from the amino acid sequence of A2 protein with dog serum (pAb). Peptides were prepared by the Spot method on cellulose membranes (Section 2) and dogs anti-A2 antibody binding (serum diluted 1:100) to cellulose-bound peptides was detected by an alkaline phosphatase-coupled anti-dog antibody (diluted 1:2000). (B) Percentage of reactivity of each peptide recognized by dog antisera A2 protein. The reactive peptides were: MKIRSVRPLVVLLVC, RSVRPLVVLLVCVAA, RPLVVLLVCVAAVLA, VVLLVCVAAVLALSA and LVCVAAVLALSASAE.

**Fig 2:**
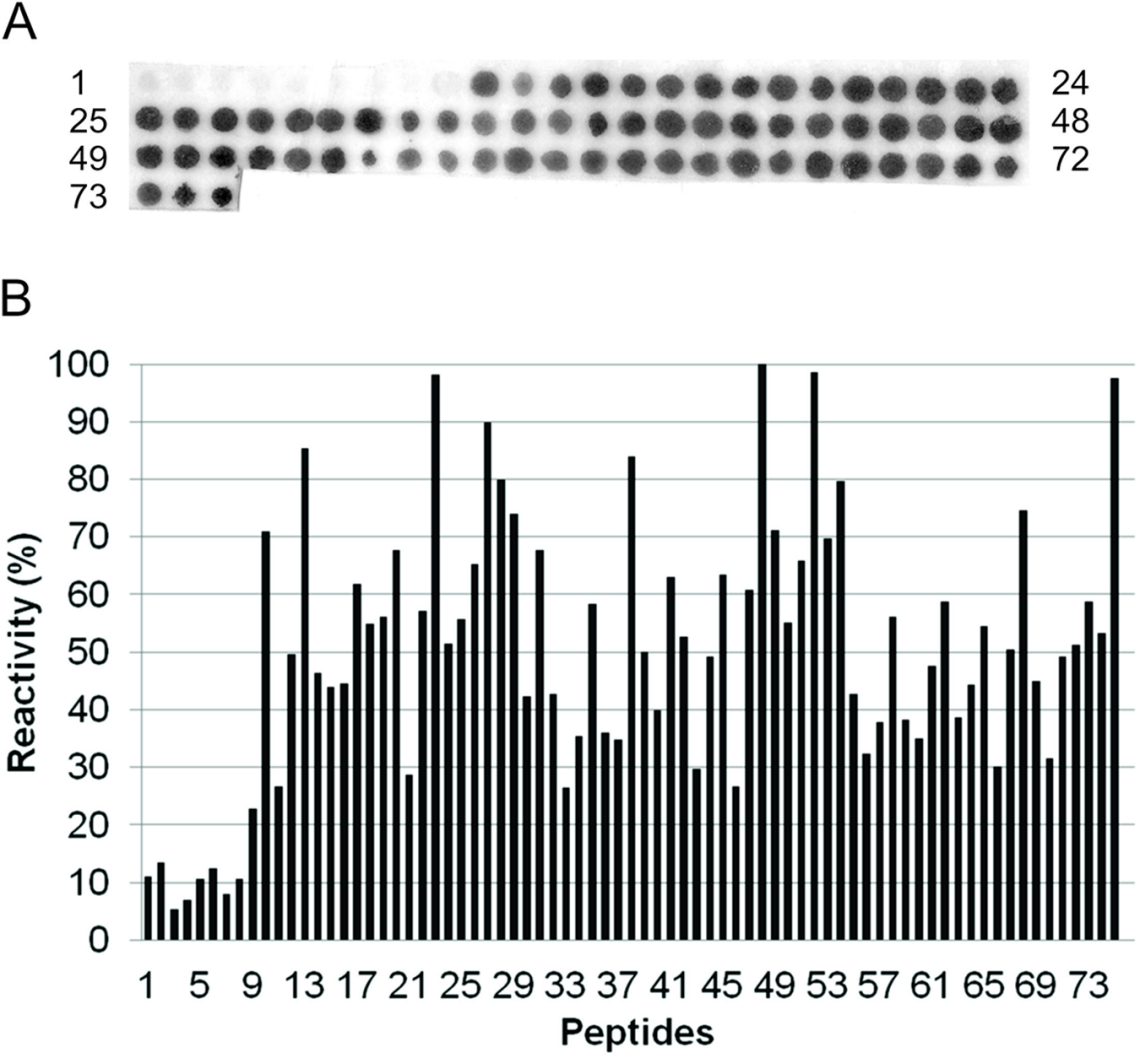
Binding pattern of a murine monoclonal antibodies with the overlapping peptides. (A) Reactivity of 15-mer overlapping peptides derived from the amino acid sequence of A2 protein with a murine monoclonal antibody (mAb). The mAb binding (concentration in 2.0 μg/mL) to cellulose-bound peptides was detected by an alkaline phosphatase-coupled anti-dog antibody (diluted 1:2000). (B) Percentage of reactivity of each peptide recognized by anti-A2 protein murine mAb. The more reactive peptide was PLSVGPQAVGPLSVG.

To map the epitopes of rec*Ld*VFA2, it was used Spot-synthesis method [19, 24]. A set of 75 overlapping peptides (15 residues, offset by 3 residues) corresponding to the complete amino acid sequence of rec*Ld*VFA2 was synthesized in a cellulose membrane (Table 2). At the end of the synthesis, peptides remain covalently bound to the membrane and can be assayed for antibody reactivity. Four different sera from dogs immunized with rec*Ld*VFA2 and an anti-rec*Ld*VFA2 monoclonal antibody were used to probe the set of peptides for antigenic reactivity. Five peptides (MKIRSVRPLVVLLVC, RSVRPLVVLLVCVAA, RPLVVLLVCVAAVLA, VVLLVCVAAVLALSA and LVCVAAVLALSASAE) corresponding to the non-repetitive N-terminal part of rec*Ld*VFA2 were strongly reactive (peptides 1–5) with all four anti- recVFA2 dog serum tested (Fig 1). Sera from non-immunized dogs were used as negative control showing no reactivity with membrane spots. When mAb anti-rec*Ld*VFA2 was used, spots corresponding to the non-repetitive N-ter region didn’t react. However, all peptides of the repetitive C-ter region showed some degree of reactivity (Fig 2). Peptide number 48 (PLSVGPQAVGPLSVG) was the more strongly reactive (Table 2). Synthetic peptides of the repetitive region containing two replicates of the amino acid sequence PLSVG (peptides number 13, 23, 38, 48 and 52) showed high reactivity with the mAb and can be considered immunodominant epitopes of the repetitive region. However, some peptides containing only a single PLSVG motif also reacted quite strongly (peptides 27, 52, 75) with mAb.

**Table 2:** Seventy-five overlapping decapentapeptides synthesized corresponding to the complete amino acid sequence of A2 *Leishmania sp* protein.

Peptide number		Peptide Sequence	Color intensity (mAb) (pAbs)		Peptide number	Peptide sequence	Color intensity (mAb) (pAbs)	
**1**	**MKIRSVRPLVVLLVC**		**11.10**	**96.13**	**39**	**VGPQSVGPLSVGPQA**	**49.95**	**20.22**
**2**	**RSVRPLVVLLVCVAA**		**13.43**	**84.25**	**40**	**QSVGPLSVGPQAVGP**	**39.85**	**22.07**
**3**	**RPLVVLLVCVAAVLA**		**5.34**	**99.79**	**41**	**GPLSVGPQAVGPLSV**	**63.11**	**20.82**
**4**	**VVLLVCVAAVLALSA**		**6.95**	**87.53**	**42**	**SVGPQAVGPLSVGPQ**	**52.71**	**16.86**
**5**	**LVCVAAVLALSASAE**		**10.62**	**80.68**	**43**	**PQAVGPLSVGPQSVG**	**29.71**	**19.54**
**6**	**VAAVLALSASAEPHK**		**12.36**	**36.52**	**44**	**VGPLSVGPQSVGPLS**	**49.13**	**25.01**
**7**	**VLALSASAEPHKAAV**		**7.94**	**26.89**	**45**	**LSVGPQSVGPLSVGP**	**63.51**	**26.16**
**8**	**LSASAEPHKAAVDVG**		**10.69**	**19.40**	**46**	**GPQSVGPLSVGPQAV**	**26.72**	**17.06**
**9**	**SAEPHKAAVDVGPLS**		**22.77**	**31.91**	**47**	**SVGPLSVGPQAVGPL**	**60.71**	**29.32**
**10**	**PHKAAVDVGPLSVGP**		**70.96**	**32.71**	**48**	**PLSVGPQAVGPLSVG**	**100.00**	**35.31**
**11**	**AAVDVGPLSVGPQSV**		**26.67**	**20.70**	**49**	**VGPQAVGPLSVGPQS**	**71.15**	**36.22**
**12**	**DVGPLSVGPQSVGPL**		**49.65**	**29.34**	**50**	**QAVGPLSVGPQSVGP**	**55.11**	**26.75**
**13**	**PLSVGPQSVGPLSVG**		**85.37**	**29.66**	**51**	**GPLSVGPQSVGPLSV**	**65.83**	**22.91**
**14**	**VGPQSVGPLSVGPQA**		**46.38**	**24.66**	**52**	**SVGPQSVGPLSVGPQ**	**98.62**	**30.74**
**15**	**QSVGPLSVGPQAVGP**		**43.93**	**22.79**	**53**	**PQSVGPLSVGPQSVG**	**69.84**	**29.47**
**16**	**GPLSVGPQAVGPLSV**		**44.48**	**25.30**	**54**	**VGPLSVGPQSVGPLS**	**79.79**	**24.82**
**17**	**SVGPQAVGPLSVGPQ**		**61.76**	**24.81**	**55**	**LSVGPQSVGPLSVGS**	**42.65**	**16.10**
**18**	**PQAVGPLSVGPQSVG**		**54.83**	**24.64**	**56**	**GPQSVGPLSVGSQSV**	**32.33**	**21.36**
**19**	**VGPLSVGPQSVGPLS**		**56.06**	**25.01**	**57**	**SVGPLSVGSQSVGPL**	**37.82**	**20.93**
**20**	**LSVGPQSVGPLSVGP**		**67.72**	**25.85**	**58**	**PLSVGSQSVGPLSVG**	**56.15**	**26.50**
**21**	**GPQSVGPLSVGPQAV**		**28.75**	**21.04**	**59**	**VGSQSVGPLSVGPQS**	**38.19**	**24.80**
**22**	**SVGPLSVGPQAVGPL**		**57.10**	**32.53**	**60**	**QSVGPLSVGPQSVGP**	**35.07**	**18.11**
**23**	**PLSVGPQAVGPLSVG**		**98.23**	**34.70**	**61**	**GPLSVGPQSVGPLSV**	**47.65**	**19.07**
**24**	**VGPQAVGPLSVGPQS**		**51.55**	**25.26**	**62**	**SVGPQSVGPLSVGPQ**	**58.78**	**21.28**
**25**	**QAVGPLSVGPQSVGP**		**55.71**	**27.91**	**63**	**PQSVGPLSVGPQSVG**	**38.64**	**21.87**
**26**	**GPLSVGPQSVGPLSV**		**65.37**	**18.03**	**64**	**VGPLSVGPQSVGPLS**	**44.45**	**23.09**
**27**	**SVGPQSVGPLSVGPL**		**89.87**	**27.24**	**65**	**LSVGPQSVGPLSVGP**	**54.45**	**23.85**
**28**	**PQSVGPLSVGPLSVG**		**79.92**	**24.14**	**66**	**GPQSVGPLSVGPQSV**	**30.19**	**18.21**
**29**	**VGPLSVGPLSVGPQS**		**74.04**	**26.81**	**67**	**SVGPLSVGPQSVGPL**	**50.46**	**25.96**
**30**	**LSVGPLSVGPQSVGP**		**42.24**	**25.79**	**68**	**PLSVGPQSVGPLSVG**	**74.70**	**31.42**
**31**	**GPLSVGPQSVGPLSV**		**67.67**	**22.16**	**69**	**VGPQSVGPLSVGPQS**	**44.99**	**20.80**
**32**	**SVGPQSVGPLSVGSQ**		**42.65**	**18.23**	**70**	**QSVGPLSVGPQSVGP**	**31.64**	**24.81**
**33**	**PQSVGPLSVGSQSVG**		**26.41**	**20.62**	**71**	**GPLSVGPQSVGPLSV**	**49.29**	**25.41**
**34**	**VGPLSVGSQSVGPLS**		**35.39**	**19.50**	**72**	**SVGPQSVGPLSVGPQ**	**51.17**	**18.40**
**35**	**LSVGSQSVGPLSVGP**		**58.37**	**25.74**	**73**	**PQSVGPLSVGPQSVD**	**58.78**	**32.72**
**36**	**GSQSVGPLSVGPQSV**		**36.10**	**21.20**	**74**	**VGPLSVGPQSVDVSP**	**53.32**	**27.61**
**37**	**SVGPLSVGPQSVGPL**		**34.83**	**24.31**	**75**	**PLSVGPQSVDVSPVS**	**97.48**	**32.82**
**38**	**PLSVGPQSVGPLSVG**		**83.90**	**27.51**				

### Diagnosis of canine leishmaniasis using synthetic bi-epitope in an ELISA format

A 34-mer recVFA2-derived synthetic peptide containing the sequence MKIRSVRPLVVLLVC linked by a Gly-Gly to the sequence PLSVGPQAVGPLSVG was chemically synthesized. Two amino acids were added, a Lys to the N-ter and a Cys to the C-ter regions, respectively. This synthetic peptide (bi-epitope) was used as antigen to coat ELISA plates, for an immune diagnosis of CVL. ELISA parameters (eg. antigen concentration, incubation times, serum dilution) were previously defined.

**Fig 3:**
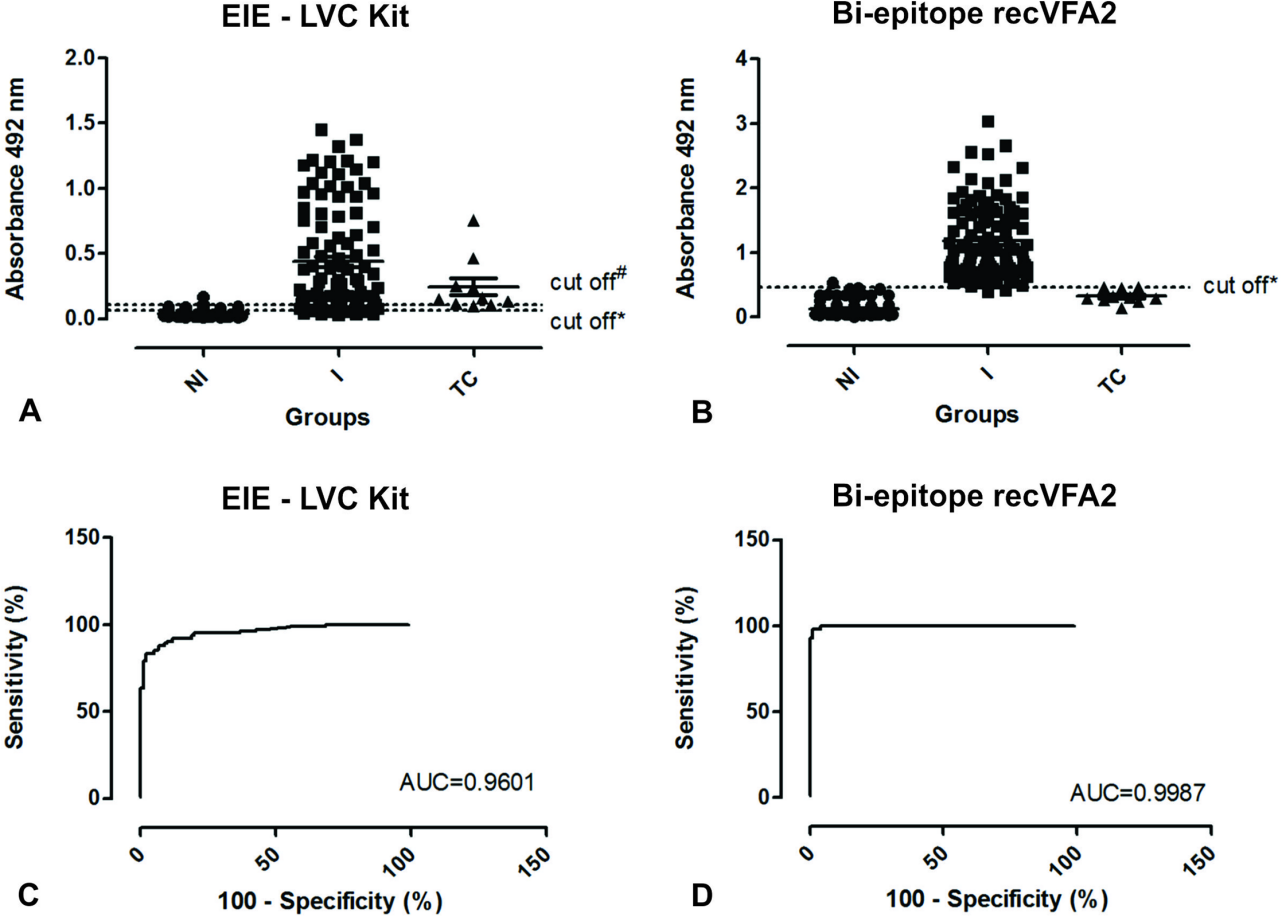
Comparison of ELISA reactivity and ROC curves obtained from canine sera against recLdVFA2 and antigen of EIE-LVC kit. **(**A) ELISA reactivity obtained from dogs sera against EIE-LVC kit. (B) ELISA reactivity obtained from dogs sera against recLdVFA2 bi-epitope. The ELISA was performed with groups of 101 uninfected dogs (NI), 101 infected with *Leishmania* (I) and 10 infected with *Trypanosoma cruzi* (TC). (C) ROC curve obtained from EIE-LVC kit. (D) ROC curve obtained from recLdVFA2 bi-epitope. The ROC curves were used to determine cut-off, sensitivity, specificity and AUC. Data variation expressed as standard error. *Cut-off obtained by ROC curve and #Cut-off obtained according to kit manufacturer.

A ready to use commercial EIE-LVC kit was included for performance comparison (Fig 3). In the conditions previously defined, the bi-epitope showed better diagnostic performance (AUC = 0.9987, 95% CI 0.9967 to 1.001; ACC = 0.9851) when compared to EIE-LVC kit (AUC = 0.9601, 95% CI 0.9351 to 0.9850; ACC = 0.9001) (Table 3). rec*Ld*VFA2 showed sensitivity (98.02%; 95% CI 93.03 to 99.76%) and specificity (99.01%; 95% CI 94,61 to 99.97%) values for detection CVL higher than those obtained with EIE-LVC kit (Se and Sp = 90.01%; 95% CI 82.54 to 95.15%). Bi-epitope ELISA was also able to discriminate *Leishmania*-infected animals from animals infected with *T*. *cruzi*, however EIE-LVC kit show cross-reactivity with *T*. *cruzi*-infected sera (Fig 3A and 3B).

**Table 3:** Diagnostic performance for rec*Ld*VFA2 and EIE-LVC kit.

	Test	
	rec*Ld*VFA2	EIE-LCV kit
Cut-off	0.4710*	0.0665*
AUC	0.9987	0.9601
ACC	0.9851	0.9010
Se (95% CI)	98.02% (93.03–99.76%)	0.9010% (82.54–95.15%)
Sp (95% CI)	99.01% (94.61–99.97%)	0.9010% (82.54–95.15%)
PPV	0.99	0.90
NPV	0.98	0.90
Kappa (95% CI)	0.97(0.937–1.00) (very good)	0.802 (0.720–0.884) (very good)^#^

*Cut-off obtained by ROC curve

#With cut-off obtained according to kit manufacturer the agreement is “good”

Agreement was calculated using parasitological assays as gold standard test.

Abbreviations: AUC: area under curve; ACC: accuracy; Se: sensitivity; Sp: specificity; PPV: positive predictive value; NPV: negative predictive value; CI: confidence interval.

## Discussion

*Ld*VFA2 protein was identified as an important candidate for vaccine development against visceral leishmaniasis [11, 13, 25].

B-cell epitope in *Ld*VFA2 was previously located within the repetitive units using sera from rec*Ld*VFA2-vaccinated mice and with the *Ld*VFA2-specific monoclonal antibody [10]. A recent study has demonstrated that humoral response to rec*Ld*VFA2 in dogs is associated, presumably, with protective immunity against *Leishmania spp*. parasites [26], however, B-cell epitope analysis with antibodies elicited in dogs were not conducted. Thus, researches for diagnosis or vaccines applied to dogs could be eventually improved by the identification of B-cell epitope mapping of *Ld*VFA2, using homologous antibodies. In this way, the present study, provided mapping of continuous B-cell epitopes, using sera from rec*Ld*VFA2 immunized dogs and a mouse monoclonal antibody (anti-rec*Ld*VFA2 mAb), by performing a systematic 15 mer peptide-scan along the complete rec*Ld*VFA2 sequence.

Anti-rec*Ld*VFA2 dog sera recognize peptides derived from the non-repetitive N-term region of rec*Ld*VFA2. Immunoreactivity of peptides bound to membranes reveals five sequences containing epitopes close to this region (peptides 1–15, 4–18, 7–21, 10–24 and 13–27). B-cell epitopes within the repetitive C-term region were not identified. On the other hand using an anti-*Ld*VFA2 mAb, B-cell epitopes in rec*Ld*VFA2 were located solely within the repetitive units, which is consistent with a previous report [27]. The two most reactive peptides have two repetitions of the sequence PLSVG (spots 23 and 48: region 67–81 and 122–135). This sequence PLSVGPQAVGPLSVG is repeated four times in the complete sequence of the *Ld*VFA2 protein. Spots 13 and 38 are also very reactive and have only one substitution in its sequence (alanine for serine), when compared to the spots 23 e 48. Thus, residues PLSVG-(X)_5_ –PLSVG are key contributors to the antigenic recognition of the peptide by specific mAb. However, spots 27 and 75 have only one sequence PLSVG and are also very reactive. We do not have a clear explanation for this observation.

A rec*Ld*VFA2 derived chimeric peptide based on mapped continuous B-cell epitopes MKIRSVRPLVVLLVC and PLSVGPQAVGPLSVG was synthesized and used in ELISA experiments for serum diagnosis of CVL. In fact, we prepared 34-mer rec*Ld*VFA2-derived peptides containing a sequence of 15 residues from one part of rec*Ld*VFA2 linked by a Gly-Gly dipeptide to a pentadeca sequence from another part of the protein, in order to bring together regions that are apart from each other in the linear sequence of the recombinant peptide. Glycine spacers’ separating the peptides increases their recognition by antibodies by providing a better exposition of chains for interaction [28]. N-ter and C-ter extremities were capped respectively by a Lysine and a Cysteine residue to allow further specific coupling to protein carriers (not used in this work). The 34-mer bi-epitope peptide was used as coating antigen in an ELISA format.

Accurate diagnosis of canine leishmaniasis is essential towards a more efficient control of this zoonosis, but is no yet achieved due to the high incidence of serological cross-reactions, mainly with other tripanosomatids antigens in canine serum samples [29]. The use of synthetic peptides [30, 31] as antigens in diagnosis of canine leishmaniasis may limit cross reactivity. It would also circumvent reliance on parasite extracts, which are not easy to reproducibly produce, and thus may help assay standardization. In the present work, encouraging results were obtained using the synthetic bi-epitope as coating antigen. Bi-epitope ELISA diagnostic test showed better sensitivity and specificity than the EIE-LVC kit, which is considered the gold standard for CVL diagnosis and high degree of agreement with parasitological techniques for the leishmaniasis diagnosis. These results using peptides selected by a peptide scanning method showed a better performance than others studies with synthetic peptides for CVL immunodiagnosis tests [32, 33, 30, 34].

In conclusion, data presented in the current study suggest that it is feasible to map B-cell epitopes from an overlapping peptide library covering the full length of rec*Ld*VFA2 and to further use the selected peptides in combination to diagnose canine visceral leishmaniasis. Further studies using sera of dogs from endemic areas (with high and low CVL prevalence) are obviously required to determine the use of these antigens for field control of CVL. Bi-epitope is derived from A2 protein and Leish-Tec vaccine is produced from this protein, so if it used in a diagnostic test of CVL, it may not be able to discriminate infected animals from those vaccinated. This antigen can be used in regions where this vaccine is not used and can also be combined with other antigenic epitopes that can minimize this fact. Finally, our work suggests that synthetic peptide-based ELISA strategy may be useful for the development of a sensitive and highly specific serodiagnosis for CVL or other parasitic diseases.

## Supporting information

S1 STARD Checklist (DOCX)

## References

[1] World Health Organization. Control of Leishmaniasis. Technical Reports Series 1990, v. 793, p. 50–52.

[2] Pace D. Leishmaniasis. J. Infect. 2014; 69(1):S10–8 25238669 10.1016/j.jinf.2014.07.016

[3] Cabral M, O´Grady JE, Gomes S, Sousa JC, Thompson H, Alexander J. The immunology of canine leishmaniosis: strong evidence for a developing disease spectrum from asymptomatic dogs. Vet. Parasitol. 1998; 76(3): 173–180. 9615951 10.1016/s0304-4017(97)00208-2

[4] Sideris V, Papadopoulou G, Dotsika E, Karagouni E. Asymptomatic canine leishmaniasis in Greater Athens area, Greece. Eur. J. Epidemiol. 1999, 15(3): 271–276. 10395058 10.1023/a:1007526401175

[5] Regina-Silva S, Fortes-Dias CL, Michalsky EM, França-Silva JC, Quaresma PF, Lima ACVMR, et al Evaluation of parasitological examination, kDNA polymerase chain reaction and rK39-based immunochromatography for the diagnosis of visceral leishmaniasis in seropositive dogs from the screening-culling program in Brazil. Rev. Soc. Bras. Med. Tropical 2014; 47(4):462–468. 10.1590/0037-8682-0064-2014 25229287

[6] Charest H and Matlashewski G. Developmental gene expression in *Leishmania donovani*: differential cloning and analysis of an amastigote-stage-specific gene. Mol Cell Biol 1994; 14: 2975–2984. 7545921 10.1128/mcb.14.5.2975-2984.1994 PMC358665

[7] Zhang WW and Matlashewski G. Loss of virulence in *Leishmania donovani* deficient in an amastigote-specific protein, A2. Proc Natl Acad Sci USA 1997; 94: 8807–8811. 9238059 10.1073/pnas.94.16.8807 PMC23140

[8] Mac Call LI and Matlashewshi G. Localization and induction of the A2 virulence factor in *Leishmania*: evidence that A2 is a stress response protein. Molecular Microbiology 2010; 77(2): 518–530. doi: 10.1111/j.1365-2958.2010.07229.x 20497497 10.1111/j.1365-2958.2010.07229.x

[9] Zhang WW, Charest H, Ghedin E, Matlashewski G. Identification and overexpression of the A2 amastigote-specific protein in *Leishmania donovani*. Mol Biochem Parasitol 1996; 78: 79–90. 8813679 10.1016/s0166-6851(96)02612-6

[10] Ghedin E, Zhang WW, Charest H, Sundar S, Kenney RT, Matlashewski G. Antibody response against a *Leishmania donovani* amastigote-stage-specific protein in patients with visceral leishmaniasis. Clin Diagn Lab Immunol 1997; 4: 530–535. 9302200 10.1128/cdli.4.5.530-535.1997 PMC170587

[11] Ghosh A, Labrecque S, Matlashewski G. Protection against *Leishmania donovani* infection by DNA vaccination: increased DNA vaccination efficiency through inhibiting the cellular p53 response. Vaccine 2001; 19: 3169–3178. 11312013 10.1016/s0264-410x(01)00023-8

[12] Zanin FH, Coelho EA, Tavares CA, Marques-da-Silva EA, Silva Costa MM, Rezende SA, et al Evaluation of immune responses and protection induced by A2 and (NH) DNA vaccines against *Leishmania chagasi* and *Leishmania amazonensis* experimental infections. Microbes Infect. 2007; 9: 1070–1077. doi: 10.1016/j.micinf.2007.05.012 17644455 10.1016/j.micinf.2007.05.012

[13] Fernandes AP, Costa MM, Coelho EA, Michalick MS, de Freitas E, Melo MN, et al Protective immunity against challenge with *Leishmania (Leishmania) chagasi* in beagle dogs vaccinated with recombinant A2 protein. Vaccine 2008; 26: 5888–5895. doi: 10.1016/j.vaccine.2008.05.095 18786587 10.1016/j.vaccine.2008.05.095

[14] Grimaldi G Jr, Teva A, Porrozzi R, Pinto MA, Marchevsky RS, Rocha MG, et al Clinical and parasitological protection in a *Leishmania infantum*-macaque model vaccinated with adenovirus and the recombinant A2 antigen. PLoS Negl. Trop. Dis. 2014; 8(6): e2853 doi: 10.1371/journal.pntd.0002853 24945284 10.1371/journal.pntd.0002853 PMC4063746

[15] Carvalho FA, Charest H, Tavares CA, Matlashewski G, Valente EP, Rabello A, et al Diagnosis of American visceral leishmaniasis in humans and dogs using the recombinant *Leishmania donovani* A2 antigen. Diagn. Microbiol. Infect. Dis. 2002; 42: 289–295. 10.1016/s0732-8893(02)00410-8 12151189

[16] Frank R. The SPOT-synthesis technique. Synthetic peptide arrays on membrane supports-principles and applications. J Immunol Methods 2002; 267: 13–26. 12135797 10.1016/s0022-1759(02)00137-0

[17] Chavez-Olortegui C, Molina F, Granier C. Molecular basis for the cross-reactivity of antibodies elicited by a natural anatoxin with alpha- and beta-toxins from the venom of Tityus serrulatus scorpion. Mol. Immunol. 2002; 38(11): 867–876. 11922945 10.1016/s0161-5890(01)00117-1

[18] Laune D, Molina F, Ferrieres G, Villard S, Bes C, Rieunier F, et al Application of the Spot method to the identification of peptides and amino acids from the antibody paratope that contribute to antigen binding. J Immunol Methods 2002; 267: 53–70. 12135800 10.1016/s0022-1759(02)00140-0

[19] Frank R. Spot-Synthesis: an easy technique for the positionally addressable, parallel chemical synthesis on a membrane support. Tetrahedron 1992; 48: 9217–9232.

[20] Merrifield RB. Solid-phase peptide synthesis.Adv. Enzimol. Relat. Areas Mol. Biol. 1969; 32: 221–296. 10.1002/9780470122778.ch6 4307033

[21] Machado de Avila RA, Stransky S, Vellosso M, Castanheira P, Schneider FS, Kalapothakis E, et al Mimotopes of mutalysin-II from Lachesis muta, snake venom induce hemorrhage inhibitory antibodies upon vaccination of rabbits. Peptide 2011, 32: 1640–1646. 10.1016/j.peptides.2011.06.028 21763377

[22] Chavez-Olortegui C, Amaral DA, Rochat H, Diniz C, Granier C. *In vivo* protection against scorpion toxins by liposomal immunization. Vaccine 1991; 9: 907–910. 1811376 10.1016/0264-410x(91)90012-u

[23] Linnet K, Bossuyt PM, Moons KG, Reitsma JB. Quantifying the accuracy of a diagnostic test or marker. Clin. Chem. 2012; 58: 1292–1301. doi: 10.1373/clinchem.2012.182543 22829313 10.1373/clinchem.2012.182543

[24] Molina F, Laune D, Gougat C, Pau B, Granier C. Improved performances of spot multiple peptide synthesis. Pept Res 1996; 9: 151–155. 8875595

[25] Coelho EA, Tavares CA, Carvalho FA, Chaves KF, Teixeira KN, Rodrigues RC, et al Immune responses induced by the *Leishmania* (*Leishmania*) *donovani* A2 antigen, but not by the LACK antigen, are protective against experimental *Leishmania* (*Leishmania*) *amazonensis* infection. Infect Immun 2003; 71:3988–3994. doi: 10.1128/IAI.71.7.3988-3994.2003 12819086 10.1128/IAI.71.7.3988-3994.2003 PMC162020

[26] Testasicca MCS, Santos MS, Machado LM, Serufo AV, Doro D, Avelar D, et al Antibody responses induced by Leish-Tec, an A2-based vaccine for visceral leishmaniasis, in a heterogeneous canine population. Veterinary Parasitology 2014; 204: 169–176. doi: 10.1016/j.vetpar.2014.04.025 24863572 10.1016/j.vetpar.2014.04.025

[27] Resende DM, Caetano BC, Dutra MS, Penido MLO, Abrantes CF, Verly RM, et al Epitope mapping and protective immunity elicited by adenovirus expressing the *Leishmania* amastigote specific A2 antigen: Correlation with IFN-γ and cytolytic activity by CD8^+^ T cells. Vaccine 2008; 26: 4585–4593. doi: 10.1016/j.vaccine.2008.05.091 18588933 10.1016/j.vaccine.2008.05.091

[28] Bialek M, Grabowski S, Kaminski Z, Kaca W. Synthetic peptides mimicking antigenic epitope of *Helicobacter pylori* urease. Acta Biochim. Pol. 2006; 53(1): 83–86. 16496040

[29] Troncarelli MZ, Camargo JB, Machado JG, Lucheis SB, Langoni H. *Leishmania* spp and/or *Trypanosoma cruzi* diagnosis in dogs from endemic and nonendemic areas for canine visceral leishmaniasis. Vet. Parasitol. 2009; 164(2–4): 118–123. doi: 10.1016/j.vetpar.2009.06.027 19625128 10.1016/j.vetpar.2009.06.027

[30] Costa MM, Penido M, Santos MS, Doro D, Freitas E, Michalick MSM, et al Improved canine and human visceral leishmaniasis immunodiagnosis using combinations of synthetic peptides in enzyme-linked immunosorbent assay. PLoS Negl. Trop. Dis. 2012; 6(5): e1622 doi: 10.1371/journal.pntd.0001622 22629475 10.1371/journal.pntd.0001622 PMC3358334

[31] Toledo-Machado CM, de Avila RA, NGuyen C, Granier C, Bueno LL, Carneiro CM, et al Immunodiagnosis of canine visceral leishmaniasis using mimotope peptides selected from phage displayed combinatorial libraries. Biomed. Res. Int. 2015; 401509. 10.1155/2015/401509 PMC4325972 25710003

[32] Faria AR, Costa MM, Giusta MS, Grimaldi G Jr, Penido MLO, Gazzinelli RT, Andrade HM. High-throughput analysis of synthetic peptides for the immunodiagnosis of canine visceral leishmaniasis. PloS Negl. Trop. Dis. 2011, 5(9): e1310a. 21931874 10.1371/journal.pntd.0001310 PMC3172188

[33] Ramos-Jesus J, Carvalho KA, Fonseca RAS, Oliveira GGS, Melo SMB, Alcântara-Neves NM, Dutra RF. A piezoelectric immunosensor for *Leishmania chagasi* antibodies in canine serum. Anal. Bioanal. Chem. 2011, 401:917–925 doi: 10.1007/s00216-011-5136-7 21667350 10.1007/s00216-011-5136-7

[34] Menezes-Souza D, Mendes TAOM, Gomes MS, Reis-Cunha JL, Nagem RAP, Carneiro CM et al Epitope mapping of the HSP83.1 protein of *Leishmania braziliensis* discloses novel targets for immunodiagnosis of tegumentary and visceral clinical forms of Leishmaniasis. Clin. Vaccine Immunol. 2014, 21(7): 917–925. 10.1128/CVI.00151-14 PMC4097446 24807053

